# Supporting dialysis policy for end stage renal disease (ESRD) in Indonesia: an updated cost-effectiveness model

**DOI:** 10.1186/s13104-022-06252-4

**Published:** 2022-12-06

**Authors:** Septiara Putri, Ryan R. Nugraha, Eka Pujiyanti, Hasbullah Thabrany, Hanifah Hasnur, Novita D. Istanti, Diah Evasari, Afiatin

**Affiliations:** 1 Health Policy and Administration Department, Faculty of Public Health, University of Indonesia, Depok, West Java Indonesia 16424; 2 Center for Health Economics and Policy Studies (CHEPS), University of Indonesia, Depok, West Java Indonesia 16424; 3 Internal Medicine Department, Faculty of Medicine, Universitas Padjajaran, Bandung, West Java Indonesia 45363

**Keywords:** Peritoneal dialysis, Hemodialysis, End stage renal disease, Cost-effectiveness

## Abstract

**Objective:**

Continuous ambulatory peritoneal dialysis (CAPD) and hemodialysis (HD) are main modalities for end stage renal disease (ESRD) patients, and those have been covered by National Health Insurance (NHI) scheme since 2014 in Indonesia. This study aims to update the cost-effectiveness model of CAPD versus HD in Indonesia setting.

**Results:**

Compared to HD, CAPD provides good value for money among ESRD patients in Indonesia. Using societal perspective, the total costs were IDR 1,348,612,118 (USD 95,504) and IDR 1,368,447,750 (USD 96,908), for CAPD and HD, respectively. The QALY was slightly different between two modalities, 4.79 for CAPD versus 4.22 for HD. The incremental cost-effectiveness ratio (ICER) yields savings of IDR 34,723,527/QALY (USD 2460).

**Supplementary Information:**

The online version contains supplementary material available at 10.1186/s13104-022-06252-4.

## Introduction

End stage renal disease (ESRD) has significantly contributed for mortality, morbidity, as well as economic impact both for patients and healthcare providers worldwide [1–3]. Due to the substantial burden of ESRD, there is a growing utilization of renal replacement therapy (RRT), including dialysis and kidney transplantation [4]. Approximately 5.4 million people are projected to receive RRT by 2030 [5]. Dialysis, is the most common treatment for ESRD, particularly haemodialysis (home or hospital-based) and peritoneal dialysis (PD) [6]. PD itself can be specified as continuous ambulatory peritoneal dialysis (CAPD) and automated peritoneal dialysis (APD).

In Indonesia, the patients’ access to dialysis in Indonesia was not high, approximately 53% with most of ESRD patients receiving HD [7]. Indonesia Renal Registry (IRR) reported that HD was the most preferable treatment, 132,142 (98%) patients compared to CAPD which were only 2478 (2%) patients [8].

More than IDR 1.5 trillion was spent in 2014, and dialysis coverage under national health insurance (NHI) system and it remains the current top substantial expense reported by BPJS Kesehatan (Indonesian health security agency) [9]. Until 2019, the government targeted a 30% first policy, CAPD however also remains underutilized among eligible ESRD patients even if it is less expensive treatment [7, 8, 10]. Therefore, we performed a cost-effectiveness model that directly compares dialysis procedures, focusing on CAPD versus hospital-based HD. We have conducted this study in early 2016, and the study indicated that CAPD was a cost-effective intervention [10]. However, there was very limited data available on parameters at that time. Hence, this study is expected to provide more updates on its cost-effectiveness results.

## Methods and materials

### Model structure

A Markov model was constructed with three mutually exclusive states: CAPD, HD, and death, performing 40 years time-horizon with annual cycle. ESRD patients’ cohort (55 years old) started into the model either receiving the CAPD or HD. The structure and assumptions of the model are presented in Additional file 1: Figure. S1.

### Patient characteristics

The patient inclusion criteria were following consecutively: (1) Adult (≥ 18 years old) (2) had confirmed ESRD diagnosis by a nephrologist, with glomerular filtration rate (GFR) < 15 ml/min (1.73 m^2^) (3) Patients who received HD or CAPD started within January 2014 to December 2015 (4) Received at least 2 outpatient dialysis treatment in similar hospital/centre. We excluded patients that (1) had been receiving various RRT (2) Different hospitals for continuing dialysis cycle for less than 3 months (3) Drop-out (discontinued), had significant gap (1 month without dialysis), or died within 3 months of dialysis procedure. Consistent with a real ratio between dialysis modalities in Indonesia, we retrieved a total sample of 110 patients (28 CAPD patients, 92 HD patients). Patient characteristics is presented in Additional file 1: Table S1.

The non-hospital and private clinics HD exist in Indonesia. However, we only gathered HD patients in the hospital since this study only focused for ESRD patients who were covered under the NHI scheme.

### Survival and transition probability

Survival data were using published literature data, since there was an absence of updates of survival analysis studies assessing both CAPD relative to HD in Indonesia. The parameter from IRR in 2007–2012 was used in previous studies as an economic model’s parameter, however it is only for HD patients [10, 11] Hence, we argued to utilize and update published literature for survival data as best as we can to represent Indonesia context..The data from the survival study were applied to the model, year 1-year 5 indicated the rates that were transformed into annual probability when running the model. We assumed the probability was constant after year 5. All input parameters were presented in Table 1.

**Table 1:** Input parameters

Parameters	Value (mean/rate)	SE	Range	Distribution	References
Survival and transition probabilities					
CAPD to HD	0.067	0.020	0.058–0.081	Beta	Surendra et al. [16]
HD to CAPD	0.007	0.002	0.002–0.001	Beta	Surendra et al. [16]
Peritonitis complication	0.200	0.010	0.180–0.220	Beta	Gupta et al. [17]
Vascular access complication	0.100	0.013	0.075–0.125	Beta	Xue et al. [18]
CAPD (survival)					
Year 1	0.800	0.006	0.788–0.812	Beta	Gunawan and Sakti [19]
Year 2	0.720	0.008	0.704–0.736	Beta	Assumed*
Year 3	0.600	0.009	0.582–0.617	Beta	Gunawan and Sakti [19]
Year 4	0.570	0.009	0.542–0.577	Beta	Assumed
Year 5	0.520	0.009	0.502–0.537	Beta	Gunawan and Sakti [19]
HD (survival)					
Year 1	0.824	0.006	0.811–0.837	Beta	Afiatin et al., [20]
Year 2	0.706	0.008	0.690–0.722	Beta	Afiatin et al., [20]
Year 3	0.621	0.009	0.604–0.638	Beta	Afiatin et al., [20]
Year 4	0.580	0.009	0.563–0.598	Beta	Afiatin et al., [20]
Year 5	0.553	0.009	0.536–0.571	Beta	Afiatin et al., [20]
Direct medical costs					
Pre-dialysis set-up_CAPD	16,010,564	165,562	15,686,062–16,335,065	Gamma	Hospital billing
Pre-dialysis set-up_HD	16,150,823	2,337,311	11,569,693–20,731,952	Gamma	Hospital billing
DMC_CAPD	142,328,780	6,008,598	135,173,358–149,484,201	Gamma	Hospital billing
DMC_HD	120,289,134	3,650,725	108,512,280–132,065,987	Gamma	Hospital billing
CC_CAPD	9,592,093	4,178,178	1,402,864–17,781,321	Gamma	Hospital billing
CC_HD	27,173,929	7,158,016	13,144,217–41,203,640	Gamma	Hospital billing
Direct non-medical costs					
DNMC_CAPD	5,266,455	1,353,606	2,613,387–7,919,522	Gamma	Interview
DNMC_HD	10,083,572	950,959	8,219,690–11,947,453	Gamma	Interview
Indirect costs					
ID_CAPD	7,196,578	1,535,788	4,186,434–10,206,722	Gamma	Interview
ID_HD	10,858,993	968,098	8,961,519–12,756,466	Gamma	Interview
Utility					
U_CAPD	0.81	0.04	0.73–0.88	Beta	Interview
U_HD	0.65	0.03	0.60–0.71	Beta	Interview
U_CAPD_com	0.31	0.09	0.13–0.39	Beta	Afiatin et al. [11],
U_HD_com	0.37	0.11	0.15–0.58	Beta	Afiatin et al. [11],
Discounting					
Cost	3%				HTA guideline [17]
Effect	3%				HTA guideline [17]

Costs are in IDR, *DMC* direct medical costs, *CC* complication cost, *DNMC* direct non-medical cost, *ID* indirect cost, *U* utility, complication.

*Due to the data reporting limitation as newly published evidence, we assumed the standard error for CAPD is similar to HD, since there were no significant differences in terms of survival results

### Costs

From a societal perspective, the costs incurred in this study include direct medical costs, direct non-medical costs, and indirect costs. Direct medical costs were collected form hospital billing data. Billing data in this study was in hospital tariff form. Furthermore, direct non-medical costs and costs related to productivity loss (indirect costs) were primarily collected by interviewing patients. Patients were interviewed before receiving dialysis in hospital or starting CAPD (direct face to face interview or by phone). The written approval and informed consent were gathered from patients Table 2.

**Table 2:** Total costs, life years gained (LYGs), quality-adjusted life years (QALYs), and incremental cost-effectiveness ratio (ICER)

	CAPD	HD	ICER/QALY
Costs	1,348,612,118	1,368,447,750	(34,723,527)
LYG	6.428	6.432	
QALY	4.79	4.22	

*ICER* incremental cost-effectiveness ratio, *LY* = life years gained, *QALY* quality adjusted life years

**Fig. 1:**
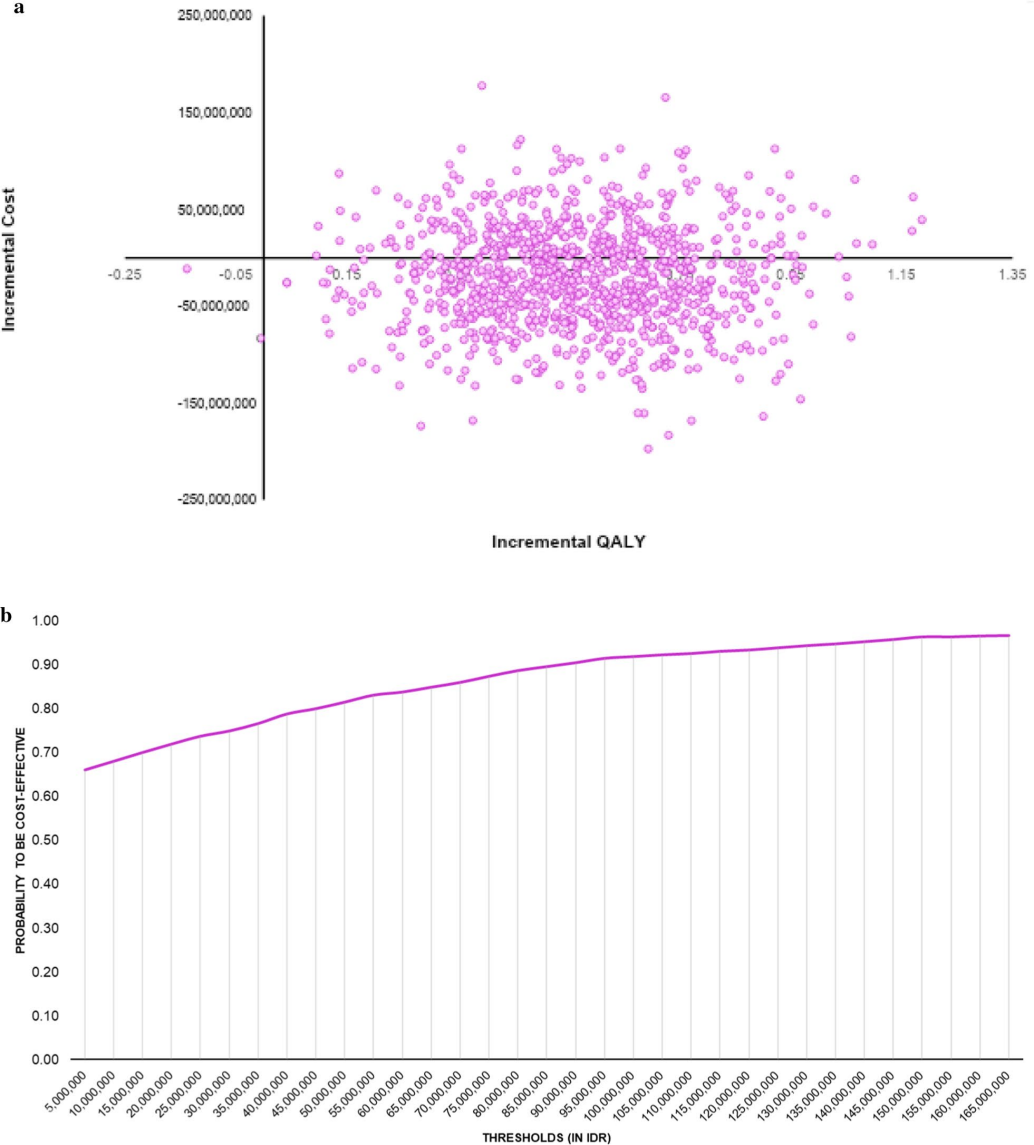
**a** cost-effectiveness plane (CE Plane). **b** cost-effectiveness acceptability curve (CEAC)

*Results are in discounted estimation. Costs are in Indonesian rupiah (IDR).One-way scenario-deterministic analysis showed that if we change several key input parameters in range 5–10% assumptions, the final ICER results indicate the good value for money for CAPD. The plots were scattered into two quadrants on CE plane, the scatter plot illustrates that as the incremental costs increased in accordance with the changes in incremental QALY (Fig. 1a), also particularly in cost saving CE plane area where there was also indicating the probability of CAPD as cost-saving, substantial QALY benefit with lower costs. Uncertainty deemed existed, particularly the wide range of the incremental QALY

To estimate the annual costs of patients, we manually calculated the monthly expenditures and then multiplied them by twelve. In practice, patients received dialysis 3 times/week, 12 per month. Since we collected data in the 2014–2015 time frame, we adjusted the costs value to 2020 IDR. We performed a 3% discount rate both for costs and effect [12].

### Utility

Quality of life (Qol) values used EuroQoL EQ-5D-3L questionnaire that has been officially translated. Patients were directly interviewed—fitted with our model states. Our previous study was using the Thailand version to convert the QoL scores into value set [13]. We finally have our Indonesian value set published in 2017 [14], however this is intended to accommodate the EQ-5D-5L questionnaire, we therefore remain to report EQ-5D-3L results using the Thai value set.

### Cost-effectiveness analysis

The economic assessment for this study applying cost utility analysis (CUA). Since Indonesia has not yet constructed the country-based cost-effectiveness threshold [12], the health technology with ICER/QALY values that was not greater than 1–3 GDP/capita was considered as cost-effective (1 GDP = IDR 54,6 million or USD 3,870) [15].

### Sensitivity analyses

One-way deterministic-scenario sensitivity analysis was performed with simulating various plausible ranges of key input parameters (Additional file 1: Table S2). A probabilistic sensitivity analysis (PSA) was also performed using monte carlo simulation with 1000 iterations simultaneously of all parameters with their respective distribution.

## Results

Patients’ characteristics were presented in Additional file 1. The result of utility data were 0.81 and 0.65, for CAPD and HD respectively. Patients with complications have utility values 0.31 for CAPD and 0.37 for HD [10].From base case analysis, the life years gained (LYG) between two modalities were not different, both having 6.43 years. In terms of QALY, CAPD showed its favourability compared to HD, 4.79 and 4.22, respectively. It was indicated due to very slight difference between survival data, however, the quality of life value was considerably different between CAPD and HD, which thus influenced the QALY results.

The average total costs showed that HD had higher costs than CAPD although CAPD itself has higher direct medical costs. This was because of the larger portion incurred in direct non-medical costs and indirect costs (particularly transportation and productivity costs). Using societal perspective, it concluded that the CAPD may save IDR 34 million (USD 2460) per QALY, compared with HD. For a long run, initial first CAPD policy for eligible ESRD patients potentially be a promising choice and have good value for money.

At the maximum defined threshold, 1–3 GDP per capita (1 GDP = IDR 54,6 million or USD 3870) besides its cost-saving result, the highest probability to be cost-effective, approximately around threshold IDR 100,000,000–165,000,000 (Fig. 1b).

## Discussions

From our analysis using societal perspective, CAPD provided good value for money, as a cost-saving treatment compared to HD. This result finally provided the most updated economic evaluation on CAPD and HD, with more representative input parameters and updated monetary values that potentially enrich evidence-based policy in Indonesia context.

This economic evaluation echoed with several studies in other countries and setting, PD provided considerable ICER results compared to HD [21, 22]. In Indian context, initial policy using PD was cost-saving compared to HD for kidney failure patients, with QALY 3.3 versus 1.6, respectively. The result of economic evaluation using societal perspective could be utilized as a base judgement for price negotiation for PD consumables in India [17]. Consistent with this finding, a study in Hongkong context confirmed that as first-policy treatment PD is a cost-saving relative to hospital-based HD with ICER USD 1195 per QALY [23]. In Finland, cost-effectiveness ratio (CER) was lower in PD than HD in four strategies on initial implementation years [24].

If compared to supportive care, PD also provided the cost-effective result in Malaysia and Singapore setting. [16, 25] The PD provided the higher clinical benefit, QALY and considerable ICER. Previous study in Indonesia, confirmed that PD exceeds the maximum threshold compared to supportive care. However, budget impact analysis estimated that PD first policy can be beneficial in terms of lower transportation and indirect costs, as well as 5 years financial impact for reimbursement policy [11].

In terms of policy implementation, there are some concerns that should be considered by decision makers such as: Making CAPD more affordable and accessible, CAPD needs to become the priority treatment for ESRD patients, and ensure the supply and capacity of CAPD together with improving of HD services in Indonesia. Moreover, the government needs to strengthen capacity and infrastructure, such as supply chain.

## Conclusions

CAPD was a cost-effective treatment compared to HD for ESRD patients in Indonesia. It must be noted that the policy impact for this study is not intended to replace or eliminate HD, vice versa. Our results provided the evidence of potential first-policy on dialysis, that is, showing its benefit and supporting rational resource allocation decision plan. The transition of dialysis and other modalities itself indeed remain justified by the specific clinical condition of ESRD patients and other complex decisions beyond this cost-effectiveness evidence.

## Limitations

First, the sample was limited, particularly in CAPD group. Although the proportion reflects the real number of samples between two groups in Indonesia on dialysis utilization (98% vs 2%, for CAPD vs HD, respectively), this may imply the uncertainty in parameters. Second, due to the lack of local survival data and clinical trials, this study preferred to use the best available data in Indonesia, until now there is no direct comparison evidence between CAPD and HD. Third, the value set used for utility parameters remains using other countries’ data, due to the fact that we did not have an Indonesian value set yet in 2016. We also recognized that the clinical characteristics in this study only based on the age, gender, and geographical distance to healthcare. Moreover, we used hospital sites focusing in Jakarta and West Java to collect costs data. As consequence, there was substantial variation in terms of hospital tariff across regions (direct medical costs) on different hospital levels as well as transportation costs and productivity loss.

## Supplementary Information

**Additional file 1. Table S1.** Patient characteristics. **Figure S1.** Schematic Markov model. **Table S2.** One-way deterministic sensitivity analysis.

## Data Availability

More detailed patient level data on costs and utility are available from info@cheps.or.id upon reasonable request. Direct medical costs data was directly obtained from hospital billings and the public access is closed. We requested the direct medical cost data under official permission from hospitals.

## Abbreviations

CAPD: Continuous ambulatory peritoneal dialysis
CKD: Chronic kidney disease
CUA: Cost utility analysis
ESRD: End stage renal disease
HD: Hemodialysis
ICER: Incremental cost effectiveness ratio
LYG: Life years gained
NHI: National health insurance
QALY: Quality adjusted life years
RRT: Renal replacement therapy
