# Supplementary material for: Supporting dialysis policy for end stage renal disease (ESRD) in Indonesia: an updated cost-effectiveness model
Source: BMC Res Notes. 2022 Dec 6;15:359. doi: 10.1186/s13104-022-06252-4 (PMC9724412; doi:10.1186/s13104-022-06252-4)
Supplement: Supplementary file 1 — Additional file 1. Table S1. Patient characteristics. Figure S1. Schematic Markov model. Table S2. One-way deterministic sensitivity analysis. [file 13104_2022_6252_MOESM1_ESM.docx]

**Table S1. Patient characteristics**

| **Variable** | **Hemodialysis (HD)** | | **CAPD** | |
| --- | --- | --- | --- | --- |
|  | **n** | **%** | **n** | **%** |
| **Gender** |  |  |  |  |
| Males | 54 | 58.7 | 17 | 60.7 |
| Females | 38 | 41.3 | 11 | 39.3 |
| **Age** |  |  |  |  |
| 15-34 years | 9 | 9.8 | 3 | 11.1 |
| 35-54 years | 39 | 42.4 | 14 | 51.9 |
| ≥ 55 years | 44 | 47.8 | 10 | 37 |
| **Hospital** |  |  |  |  |
| Cikini hospital | 19 | 20.7 | N/A | N/A |
| Hasan Sadikin hospital | 60 | 65.2 | 19 | 67.8 |
| Tarakan hospital | 13 | 14.1 | 9 | 32.1 |
| **Distance from home to receive dialysis services** |  |  |  |  |
| 6-10 km | 3 | 3.5 | 1 | 3.8 |
| 11-15 km | 5 | 5.9 | 25 | 96.2 |
| ≥16 km | 77 | 90.6 | N/A | N/A |
| **Potential income loss by job**  Patients with professional activities | 55 | 60 | 15 | 54 |
| Companion/caregiver with professional activities | 24 | 25 | 12 | 43 |

**Figure S1. Schematic Markov model**


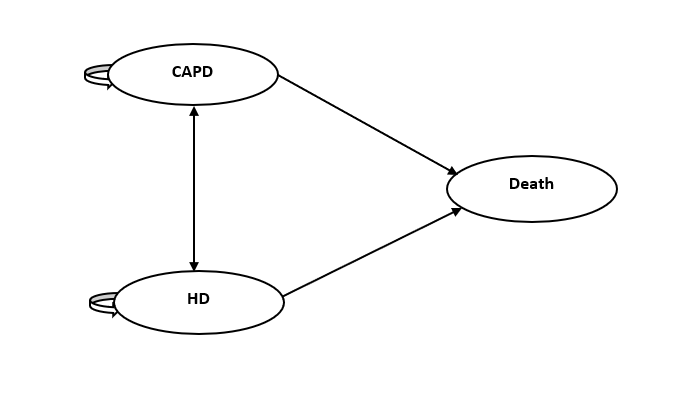


Three mutually exclusive states in Markov model: CAPD, HD, and death, performing 40 years time-horizon with annual cycle. ESRD patients’ cohort (55 years old) started into the model either receiving the CAPD or HD, with assumption the stable transition probability on these initial states (Table 1). Patients may remain in each modality for some time, or move (with the assumption of potential complication or clinical circumstances due to dialysis) to another modality or die. 3% discount rate was applied both for costs and benefits. A half-cycle correction was applied to the model as well.

**Table S2. One-way deterministic sensitivity analysis**

| **Parameters tested** | **Costs (IDR)** | | **QALY** | | **Changes in ICER (%)** |
| --- | --- | --- | --- | --- | --- |
|  | **CAPD** | **HD** | **CAPD** | **HD** |  |
| ***Base case*** | 1,348,612,118 | 1,368,447,750 | 4.79 | 4.22 | (34,723,527) |
| ***Scenario 1*** | 1,516,084,512 | 1,532,611,398 | 5.02 | 4.43 | (28,434,772)  +18% |
| CAPD to HD (+5%) |  |  |  |  |  |
| HD to CAPD (+20%) |  |  |  |  |  |
| DMC_CAPD (+15%) |  |  |  |  |  |
| DMC_HD (+15%) |  |  |  |  |  |
| CC_CAPD (+15%) |  |  |  |  |  |
| CC_HD (+15%) |  |  |  |  |  |
| U_CAPD (+5%)  U_HD (+5%) |  |  |  |  |  |
| ***Scenario 2*** | 1,237,650,758 | 1,258,598,418 | 4.53 | 4.02 | (40,888,411)  -17% |
| CAPD to HD (+5%) |  |  |  |  |  |
| HD to CAPD (+30%) |  |  |  |  |  |
| DMC_CAPD (-10%) |  |  |  |  |  |
| DMC_HD (-10%) |  |  |  |  |  |
| CC_CAPD (-10%) |  |  |  |  |  |
| CC_HD (-10%) |  |  |  |  |  |
| U_CAPD (-5%) |  |  |  |  |  |
| U_HD (-5%) |  |  |  |  |  |
| Discount rate (5%, both costs, and effect) |  |  |  |  |  |

The plausible changes in deterministic scenario analysis were applied. In scenario 1, we applied the range values by following expert panel (clinicians, epidemiologists, and health economists) discussion and inputs during this research completed in 2016. For example, to make the shift from HD to CAPD (20%-30%), due to the target to make the higher gradual CAPD utilization in Indonesia. The costs were changed also with the assumption that might be different tariffs applied in different types of hospitals/regions (i.e: type A, type B hospitals). In terms of utility/quality of life, we acknowledged the limitation of samples collected or bias that might occur during direct patient interviews.

Scenario 2. showed a similar assumption in terms of dialysis utilization. However, in the worst-case scenario since we use tariff (margin exists) instead of direct unit cost and resource use calculation. The discount rate was assumed, at that time we did not have a national guideline for the economic evaluation of health technologies, so we performed this scenario.
